# The Shakespeare Myth

**Published:** 1913-03

**Authors:** 


### The Shakespeare Myth

Sir Edwin Durning Lawrence incloses the following extract from the Argument of Tragi-comedy of Octavia, done by Samuel Brandon in 1598, to show how firmly Bacon had established the English language at that time, and how little change has since occurred. "After the death of Julius Caesar and the overthrow of Brutus and Cassius, the chief conspirators, the Government of the Roman Empire remained unto Octavius Caesar, Marke Anthony, and (at that time) Sextus Pompeius, Marke Anthony, to confirm an inviolable league of amitie, between Caesar and himselfe, tooke to wife Octavia, the sister of Caesar. Anthony and Caesar, falling at debate, met at Tarentum with their armies, and had bin the cause of much bloodshed; but they were appeased by the wisdome of Octavia." . . . . . .

Sir Edwin further corrects our impression that Shakespeare was accepted by London society or was highly regarded in Stratford, and states that "Among the large number of letters in existence, there is not one that even mentions that the writer had ever seen or heard of W. Shakespeare of Stratford." We regret that lack of space, owing to pressure of more strictly medical matter, prevents our publishing the whole of Sir Edwin's letter.

